# Evaluating the Ability of Artificial Intelligence to Address Nuanced Cardiology Subspecialty Questions: ChatGPT and CathSAP

**DOI:** 10.1016/j.jscai.2025.102563

**Published:** 2025-03-18

**Authors:** Saumya Nanda, Khaled Abaza, Pyae Hein Kyaw, Robert Frankel, Partha Sardar, Sahil A. Parikh, Tharun Shyam, Saurav Chatterjee

**Affiliations:** a Department of Internal Medicine, Maimonides Medical Center, Brooklyn, New York; b Department of Cardiology, Maimonides Medical Center, Brooklyn, New York; c Maimonides Heart & Vascular Institute, Brooklyn, New York; d Interventional Cardiology, Columbia University Irving medical Center, New York, New York; e Division of Cardiovascular Medicine, Zucker School of Medicine at Hofstra/Northwell, Hempstead, New York; f NYU Langone/Tisch Hospitals, New York, NY

**Keywords:** artificial intelligence, Cath Self Assessment Program, ChatGPT, medical education

## Abstract

**Background:**

Recent developments in artificial intelligence (AI), particularly in large language models, have shown promise in various fields, including health care. However, their performance on specialized medical board examinations, such as interventional cardiology assessments, remains relatively unexplored.

**Methods:**

A cross-sectional study was conducted using a data set comprising 360 questions from the Cath Self Assessment Program (CathSAP) question bank. This study aimed to assess the overall performance of Chat Generative Pre-trained Transformer (ChatGPT) and compare it to that of average test takers. Additionally, the study evaluated the impact of pertinent educational materials on ChatGPT’s responses, both before and after exposure. The primary outcome measures included ChatGPT’s overall percentage score on the CathSAP examination and its performance across various subsections. Statistical significance was determined using the Kruskal-Wallis equality-of-populations rank test.

**Results:**

Initially, ChatGPT achieved an overall score of 54.44% on the CathSAP exam, which improved significantly to 79.16% after exposure to relevant textual content. The improvement was statistically significant (*P* = .0003). Notably, the improved score was comparable with the average score achieved by typical test takers (as reported by CathSAP). ChatGPT demonstrated proficiency in sections covering basic science, pharmacology, and miscellaneous topics, although it struggled with anatomy, anatomic variants, and anatomic pathology questions.

**Conclusions:**

The study demonstrates ChatGPT’s potential for learning and adapting to medical examination scenarios, with a notable enhancement in performance after exposure to educational materials. However, limitations such as the model’s inability to process certain visual materials and potential biases in AI models warrant further consideration. These findings underscore the need for continued research to optimize the use of AI in medical education and assessment.

## Introduction

The Interventional Cardiology Board Examination is a crucial milestone and is often a prerequisite for practicing interventional cardiology in the United States. This exam is designed to assess a candidate’s comprehension, decision-making prowess, and clinical acumen for adept interventional cardiology practice.^1^

Trainees often use the Cath Self Assessment Program (CathSAP) to prepare for these demanding questions. Developed jointly by the American College of Cardiology and Society for Cardiovascular Angiography & Interventions, CathSAP offers an online question bank comprising multiple-choice questions meticulously crafted to mirror the format of board-style questions. Through this resource, trainees can hone their skills and familiarize themselves with the intricacies of interventional cardiology examination questions, ensuring thorough preparation.^2^

There is a growing body of research focused on evaluating the performance of Chat Generative Pre-trained Transformer (ChatGPT) in medical examination contexts. ChatGPT has demonstrated the ability to pass the American Heart Association’s Basic Life Support and Advanced Cardiac Life Support exams. Notably, this proficiency was confined to formats involving open-ended queries rather than multiple-choice questions, a distinction possibly rooted in ChatGPT’s inherent aptitudes that are advantageous for open-response contexts.^3^ A study by Kung et al^4^ revealed that ChatGPT achieved scores close to passing on the United States Medical Licensing Examination. The study underscored that ChatGPT’s inaccuracies stemmed primarily from a lack of information, resulting in diminished clarity and indecisiveness in the responses of artificial intelligence (AI) rather than a propensity to select incorrect answers. Although it did not achieve a passing score in the ophthalmology board-style questions, it did demonstrate a near-passing score in the radiology board-style questions.^5^^,^^6^ Comparisons between ChatGPT-4 and ChatGPT-3.5 favored the performance of 4, as was evidenced in the study evaluating the performance of the 2 large language models (LLM) on neurology and neurosurgery board-style questions.^7^^,^^8^ ChatGPT has also demonstrated proficiency in passing more advanced subspecialty examinations, such as the European Examination in Core Cardiology.^9^ More recently, an article evaluated ChatGPT’s (version 4) performance on a random selection of 60 questions from CathSAP and discovered that ChatGPT answered 61.7% of the questions correctly.^10^

In this study, we evaluated ChatGPT’s capacity to learn from user-generated prompts, with a particular focus on its ability to assimilate and apply feedback and updates. Specifically, we assessed ChatGPT’s initial competency in addressing questions from CathSAP—a widely used board review resource for interventional cardiologists and trainees—and subsequently examined its potential for generative learning when provided with pertinent text.^11^ Building on the foundation of previous studies that primarily evaluated ChatGPT’s static performance, our work shifted the focus to adaptability. Unlike earlier research, which assessed ChatGPT’s fixed capabilities, we investigated whether iterative learning and targeted training can enhance its accuracy and reasoning. By exploring its ability to integrate user feedback and domain-specific updates, this study aimed to determine the extent to which ChatGPT can evolve as a dynamic and adaptive educational tool.

## Materials and methods

This study utilized the CathSAP question bank comprised 455 questions across 10 distinct subsections. For this analysis, multimedia elements such as images and videos were excluded, resulting in a pool of 360 questions suitable for evaluation. Each question was systematically input into the ChatGPT interface based on the GPT-3.5 model (accessible via https://chat.openai.com/; version date: February 13, 2023; developed by OpenAI). The process involved the verbatim entry of questions into the ChatGPT system, followed by a meticulous comparison of the generated responses with the official answers provided by CathSAP.

The board exam blueprint delineates the distribution of questions across various subsections as follows: Case Selection and Management (20%), Procedural Techniques I and II (20%), Complications of Coronary Intervention (8%), Catheter-Based Management of Noncoronary Disease (13%), Basic Science (6%), Anatomy, Anatomic Variants, Anatomic Pathology (6%), Pharmacology (12%), Cardiac Imaging and Assessment (9%), and Miscellaneous (6%).

In instances in which ChatGPT’s responses were ambiguous, an additional directive—“Select the single-most appropriate response”—was appended to the query, in keeping with current trends in American board exam questions. To mitigate the potential influence of contextual learning from previous queries, each question was processed in a separate chat box, aligning with methodologies adopted in a comparable research study.^4^

Responses identified as incorrect were carefully recorded and subjected to a secondary round of analysis through ChatGPT. In cases in which repeated attempts yielded incorrect answers, a targeted intervention was implemented. This involved providing ChatGPT with contextually relevant textual information from the “Key Point” section of the corresponding CathSAP question to enhance the learning model’s accuracy.

ChatGPT’s performance was assessed by determining the percentage of correct answers both before and after it received the relevant “teaching” and then averaging these values across all subsections. After this process, ChatGPT’s response accuracy was benchmarked against the average test taker’s performance. The average performance of test takers was determined by calculating the mean percentage of correct responses for each question across all participants, as provided by CathSAP. Subsequently, the statistical significance of any observed disparities between ChatGPT before and after providing relevant text was evaluated using the Kruskal-Wallis equality-of-populations rank test.

## Results

ChatGPT initially achieved an overall score of 54.44% on the CathSAP exam. The question bank requires a minimum score of 70% to earn credits toward Certified Medical Education points; thus, the passing threshold was considered 70%. In comparison, the average score among question bank users was calculated as 75.81%. Although ChatGPT did not meet the overall passing criteria, it demonstrated proficiency in the Basic Science and Miscellaneous sections, achieving passing scores. Additionally, it attained a score of 66.67% in the Pharmacology section. Notably, ChatGPT’s performance exceeded that of the average test taker in the Miscellaneous section (Table 1, Central Illustration).

**Table 1:** Comparison of ChatGPT’s performance on CathSAP topics before and after “teaching.”

CathSAP topics	Performance of ChatGPT	After “teaching” ChatGPT	Average test taker	*P*
Case Selection and Management	51.42%	72.80%	79.57%	.0143
Procedural Techniques I	46.43%	89.28%	78.57%	.0013
Procedural Techniques II	47.06%	82.35%	77.74%	<.0001
Complications of Coronary Intervention	50%	79%	72.21%	.0496
Basic Science	72.22%	88.88%	85.66%	.4018
Catheter-Based Management of Noncoronary Disease	41.66%	75.00%	76.80%	.0080
Anatomy, Anatomic Variants, Anatomic Pathology Chapters	41.66%	58.33%	78.32%	.6843
Pharmacology	66.67%	79.49%	79.88%	.3073
Cardiac Imaging and Assessment	61.29%	77.42%	80.64%	.2704
Miscellaneous	76.67%	86.67%	75.30%	.5062

*P* values were calculated using the Fisher exact test. The average scores of the test takers are included for reference.

**Central Illustration:**
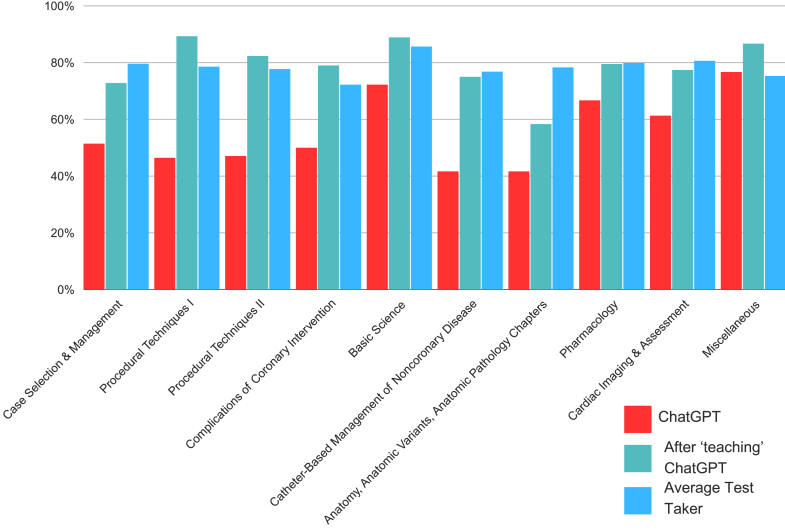
**Performance of ChatGPT compared to the average test taker before and after “teaching” ChatGPT.** This illustration compares ChatGPT’s performance with that of the average test taker across various domains of medical knowledge. The x-axis lists the domains, and the y-axis shows percentage scores. Red bars represent ChatGPT’s initial performance, green bars show ChatGPT’s performance after additional “teaching,” and blue bars depict the average test taker’s scores.

After providing the relevant text in an attempt to “teach” ChatGPT, the LLM demonstrated a significant improvement in its performance, with an overall score of 79.16%, exceeding the overall score of the average test taker, which is 75.81% (Table 1, Central Illustration). It passed all subsections except one: Anatomy, Anatomic Variants, and Anatomic Pathology chapters. However, it outperformed the average test taker in the subsections of Procedural Techniques I and II, Complications of Coronary Intervention, Basic Science, and the Miscellaneous. The difference in performance before and after being taught was statistically significant (*P* = .0003). Furthermore, no statistical difference was noted between the performance of ChatGPT after teaching and that of the average test taker (*P* = .65).

## Discussion

While earlier iterations of AI have predominantly relied on deep learning models engineered to discern and internalize data patterns, LLM emerge as a distinct category of AI algorithm proficient in predicting word sequences by drawing on contextual cues from preceding words. Consequently, when LLM undergo training with extensive text data, they can generate novel word sequences not previously encountered by the model yet are linguistically plausible.^12^ This capability has sparked growing academic interest in assessing ChatGPT’s performance in medical examination scenarios. While previous research has evaluated ChatGPT's performance on general medical examinations, there is a significant gap in understanding its ability to handle highly specialized medical content that is not publicly accessible, such as the CathSAP question bank used in cardiology.

In this study, we assessed the performance of ChatGPT-3.5, an LLM, in answering specialized cardiology questions extracted from the subscriber-exclusive CathSAP question bank (version 5, updated February 2024), thereby minimizing the likelihood of prior exposure. Our primary objective was to determine the model’s ability to generate accurate responses using publicly available information, focusing on baseline accuracy, potential improvements through tailored “teaching” interventions, and identification of strengths and limitations. The model’s accuracy improved significantly after these interventions, increasing from 54.44% to 79.16%.

Our findings indicate that targeted guidance can substantially enhance ChatGPT’s accuracy in answering highly specialized medical questions. The significant improvement observed aligns with findings such as those of Gilson et al^13^ showing that providing an “Attending Tip” to United States Medical Licensing Examination-style questions led to better performance. Specifically, ChatGPT demonstrated strong performance in pharmacology-related sections, likely due to the structured and widely accessible nature of pharmacological data in public data sets. Improved results in the Basic Sciences and Miscellaneous sections may reflect the lower complexity of these questions and the extensive availability of foundational science knowledge. ChatGPT’s poor performance in the anatomical subsection, even after tailored educational interventions, possibly stems from its difficulty grasping complex spatial relationships and subtle anatomical distinctions that are not easily conveyed through text alone. This potentially highlights an inherent limitation in the model’s capacity to handle visually oriented or spatially nuanced medical content. We hypothesize that ChatGPT’s limitations in answering questions in the clinical care-related sections stem from its reliance on algorithms and publicly available information, which may exclude content behind paywalls. Effective clinical decision making often requires integrating multiple resources and recognizing nuanced clinical findings and cues present in patient scenarios. This capability is typically honed by fellows through extensive clinical exposure, a dimension currently lacking in ChatGPT.

Comparing our results with those of other studies highlights both the potential and limitations of ChatGPT. For instance, Alexandrou et al^10^ evaluated the performance of ChatGPT-4.0 on CathSAP questions and reported an average score of 76.7% after requiring justification of incorrect answers. Although our study focused on ChatGPT-3.5, the differences in performance emphasize the importance of version-specific optimization. In contrast to the study by Alexandrou et al,^10^ which evaluated ChatGPT 4.0’s baseline performance on a limited question set without tailored guidance, our manuscript demonstrates that targeted “teaching” interventions can significantly improve ChatGPT-3.5’s accuracy. Notably, in both studies, human fellows consistently outperformed ChatGPT, underscoring the current limitations of AI in replicating expert-level clinical reasoning.

This study has some limitations. At the time of testing, ChatGPT-3.5 could not process images and video-based elements. Thus, a significant limitation of ChatGPT-3.5 identified in this study was its inability to process image- or video-based queries, which are crucial in disciplines such as interventional cardiology. To augment ChatGPT’s responses, supplementary text was incorporated from the “Key Point” sections of the questions. This supplementary material included information from relevant chapters, along with justifications for the correct answers. However, it is important to acknowledge that this additional content did not consistently address the informational gaps in ChatGPT’s initial responses. Furthermore, the supplementary learning material often failed to explicitly clarify the reasoning behind choosing one answer over the alternatives. An additional pertinent consideration is a growing concern that the utilization of similar AI models might propagate existing biases. This apprehension stems from the understanding that the data sets for training these models are complex and necessitate a more comprehensive and nuanced evaluation than in their existing forms.^14^ Another limitation of this study is the underlying assumption that the explanations provided by CathSAP are accurate without considering the possibility that ChatGPT might be correct in certain instances in which discrepancies arise.

Despite these challenges and limitations, the study underscores the transformative potential of LLM like ChatGPT, especially when combined with targeted interventions and specialized data sets. The ability to improve performance through tailored interventions suggests that LLM can become valuable tools for medical trainees, assisting in bridging knowledge gaps and reinforcing complex concepts. Creating specialized training data sets with expert clinical reasoning and case-based scenarios and addressing biases in AI systems can enhance models’ decision making.^15^ This could be particularly beneficial in resource-limited settings, where access to specialized medical education may be limited.^16^ As these models continue to evolve, they could play a pivotal role in supporting clinical workflows by aiding in evidence synthesis, guideline adherence, and case-based reasoning.^17^^,^^18^ This integration has the potential to democratize access to medical knowledge, enhance personalized medical education, and improve patient care globally.

## Conclusion

In an extensive assessment of ChatGPT’s efficacy on CathSAP interventional cardiology examination queries, this study demonstrated a notable enhancement in the AI’s performance, increasing from an initial 54.44% to 79.16% after exposure to pertinent educational materials. Of note, the improved score is comparable to the average score achieved by typical test takers. With notable proficiency in the Pharmacology, Basic Science, and Miscellaneous sections, ChatGPT’s improvement underscores its adaptability and learning capabilities. However, the study also highlighted certain limitations, including ChatGPT’s inability to process visual materials and potential biases in AI models due to the nature of their training data. These findings raise important considerations about the use of AI in medical education and assessment, emphasizing the need for careful analysis of AI’s capabilities and limitations to optimize utility.
